# Estimation of Genetic Relationships Between Individuals Across Cohorts and Platforms: Application to Childhood Height

**DOI:** 10.1007/s10519-015-9725-7

**Published:** 2015-06-03

**Authors:** Iryna O. Fedko, Jouke-Jan Hottenga, Carolina Medina-Gomez, Irene Pappa, Catharina E. M. van Beijsterveldt, Erik A. Ehli, Gareth E. Davies, Fernando Rivadeneira, Henning Tiemeier, Morris A. Swertz, Christel M. Middeldorp, Meike Bartels, Dorret I. Boomsma

**Affiliations:** Department of Biological Psychology, VU University Amsterdam, Van der Boechorststraat 1, 1081BT Amsterdam, The Netherlands; EMGO+ Institute for Health and Care Research, VU University Medical Center, Van der Boechorststraat 7, 1081BT, Amsterdam, The Netherlands; Neuroscience Campus Amsterdam, De Boelelaan 1085, 1081HV Amsterdam, The Netherlands; Generation R Study Group, Erasmus Medical Center, Rotterdam, The Netherlands; Department of Child & Adolescent Psychiatry, Erasmus Medical Center, Sophia Children’s Hospital, Rotterdam, The Netherlands; Department of Epidemiology, Erasmus Medical Center, Rotterdam, The Netherlands; Department of Internal Medicine, Erasmus Medical Center, Rotterdam, The Netherlands; School of Pedagogical and Educational Sciences, Erasmus University Rotterdam, Rotterdam, The Netherlands; Avera Institute for Human Genetics, Sioux Falls, SD USA; Department of Genetics, University Medical Center Groningen, University of Groningen, Groningen, The Netherlands; Genomics Coordination Center, University Medical Center Groningen, University of Groningen, Groningen, The Netherlands; Department of Child and Adolescent Psychiatry, GGZ inGeest/VU University Medical Center, Amsterdam, The Netherlands

**Keywords:** Genotyping platform, Heterogeneity, Imputation, GCTA, SNP-heritability, Height

## Abstract

Combining genotype data across cohorts increases power to estimate the heritability due to common single nucleotide polymorphisms (SNPs), based on analyzing a Genetic Relationship Matrix (GRM). However, the combination of SNP data across multiple cohorts may lead to stratification, when for example, different genotyping platforms are used. In the current study, we address issues of combining SNP data from different cohorts, the Netherlands Twin Register (NTR) and the Generation R (GENR) study. Both cohorts include children of Northern European Dutch background (N = 3102 + 2826, respectively) who were genotyped on different platforms. We explore imputation and phasing as a tool and compare three GRM-building strategies, when data from two cohorts are (1) just combined, (2) pre-combined and cross-platform imputed and (3) cross-platform imputed and post-combined. We test these three strategies with data on childhood height for unrelated individuals (N = 3124, average age 6.7 years) to explore their effect on SNP-heritability estimates and compare results to those obtained from the independent studies. All combination strategies result in SNP-heritability estimates with a standard error smaller than those of the independent studies. We did not observe significant difference in estimates of SNP-heritability based on various cross-platform imputed GRMs. SNP-heritability of childhood height was on average estimated as 0.50 (SE = 0.10). Introducing cohort as a covariate resulted in ≈2 % drop. Principal components (PCs) adjustment resulted in SNP-heritability estimates of about 0.39 (SE = 0.11). Strikingly, we did not find significant difference between cross-platform imputed and combined GRMs. All estimates were significant regardless the use of PCs adjustment. Based on these analyses we conclude that imputation with a reference set helps to increase power to estimate SNP-heritability by combining cohorts of the same ethnicity genotyped on different platforms. However, important factors should be taken into account such as remaining cohort stratification after imputation and/or phenotypic heterogeneity between and within cohorts. Whether one should use imputation, or just combine the genotype data, depends on the number of overlapping SNPs in relation to the total number of genotyped SNPs for both cohorts, and their ability to tag all the genetic variance related to the specific trait of interest.

## Introduction

Before embarking on Genome Wide Association (GWA) projects, the heritability of complex traits is often assessed in twin and family studies, or, more recently, assessed based on common single nucleotide polymorphisms (SNPs). Such SNP-based heritability can be estimated when genetic similarities between distantly related individuals are summarized in a genetic relatedness matrix, which then is used to predict their phenotype similarity (Visscher et al. 2010; Lubke et al. 2012; Lee et al. 2012; Zaitlen et al. 2013). This technique, known as genomic-relatedness-matrix restricted maximum likelihood (GREML; Benjamin et al. 2012), is implemented, for example, in the software package GCTA (Genome-wide Complex Trait Analysis; Yang et al. 2011). Estimating the heritability based on measured SNPs requires the availability of raw genotype and phenotype data. Therefore, these analyses are usually performed in one, or a few separate cohorts that contribute to a meta-analysis GWAS. However, in single studies, these SNP-based heritability estimates tend to have large standard errors due to small sample sizes. The large standard errors also result in variation in estimates between different studies for the same trait.

Here we investigate the possibility to combine individual-level genotype data across cohorts in order to obtain a larger and better GRM. A cross-cohort GRM will allow inclusion of all possible combinations of pairs of individuals, both within, as well as between cohorts, and estimation of the genetic variance explained by common variants (SNP-heritability) will likely improve. However, it requires sharing and pooling of raw phenotype and genotype data from multiple cohorts. For genotype data this likely means that data of multiple genotyping platforms need to be combined and this might lead to biased results due to “platform stratification”, when relationships between individuals of different cohorts are estimated based on overlapping SNPs only. In case of GWA meta-analyses, each individual cohort performs its own imputation using a reference set (e.g. HapMap or 1000 Genome) and statistical analysis prior to the combination of results. In this way the confounding effects of genotyping platforms are avoided. SNPs showing platform stratification effects will be detected with heterogeneity testing and meta-analysis Quality Control (QC). With GREML analyses, the genotyped data of cohorts need to be combined at the SNP level. If different platforms have been used for genotyping, a cross-platform imputation is required in order to combine genotypes from several cohorts and assure that all individuals have the same SNP information to estimate relationships between them.

**Fig. 1:**
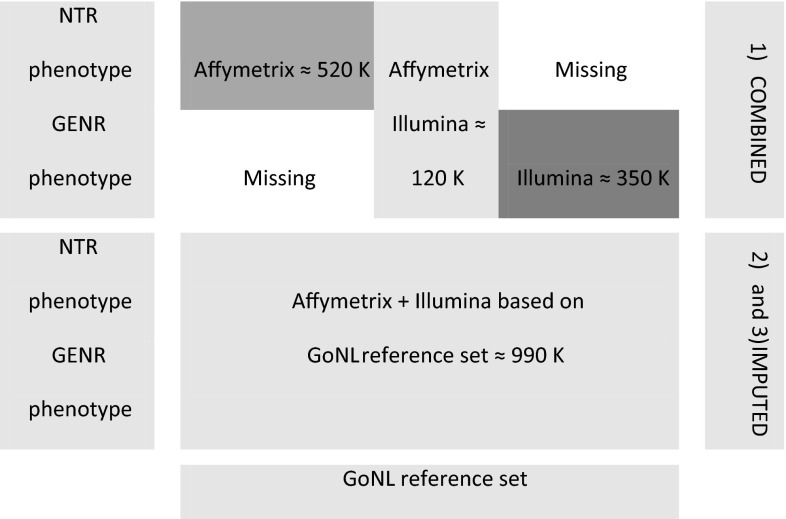
Strategies of combing two cohorts genotyped on different platforms, when two cohorts are either (*1*) combined or (*2*) and (*3*) cross-platform imputed

In this paper, we compare approaches that combine autosomal genotype data from different cohorts and genotyping platforms into a single GRM. We aim to address and resolve problems of stratification when cohorts differ in genotyping strategies and phenotype characteristics. Therefore this study has two aims: (1) to allow the combination of genetic data from two cohorts, where participants are genotyped on different platforms with little overlap, (2) to explore the effect of three different strategies of combining such data on SNP-heritability estimates, when two cohorts are either cross-platform imputed (post- or pre-combined) or just combined (Fig. 1). We base our analysis on genotype data from two Dutch cohorts, the Netherlands Twin Register (NTR; Boomsma et al. 2006; van Beijsterveldt et al. 2013) and the Generation R study (GENR; Tiemeier et al. 2012; Jaddoe et al. 2012). NTR recruits twin families across the Netherlands, whereas GENR targets a birth cohort from Rotterdam. The cohorts have genotyped their participants on different Affymetrix and Illumina platforms, respectively. We illustrate the imputation approaches and test their performance using principal components analysis (PCA) to check for stratification due to genotyping platform. Subsequently we demonstrate the differences of using cross-platform imputation versus just combining datasets for childhood height.

The methods considered to pre-combine and cross-platform impute the NTR and GENR genotype data include combining both genotype data sets at the SNP level and then phasing (i.e. estimating haplotypes) the combined data as a single dataset. We phase combined data without- and with a reference imputation set using MaCH (Li et al. 2010) and MaCH-Admix (Liu et al. 2013) and inherently impute. When a reference set was used, the data were imputed with reference to data from the Genome of the Netherlands (GoNL) project (Boomsma et al. 2014, Genome of the Netherlands Consortium 2014). The GoNL imputation reference set is a resource of sequenced data from the Netherlands, where a group of 250 trio’s from all Dutch provinces was sequenced at a depth of ~12–13×. We chose this reference panel, because this set is the closest to both cohorts with respect to their genetic background (Deelen et al. 2014). Our results show that phasing without a reference set is not able to eliminate differences between platforms. However, phasing together with a reference set helps to bring the two cohorts together with minimum platform stratification left. Strict imputation quality control (pre- and post-QC) as well as GCTA specific quality control is required to eliminate remaining platform stratification in cross-platform imputed dataset.

## Materials and methods

### Sample

**Fig. 2:**
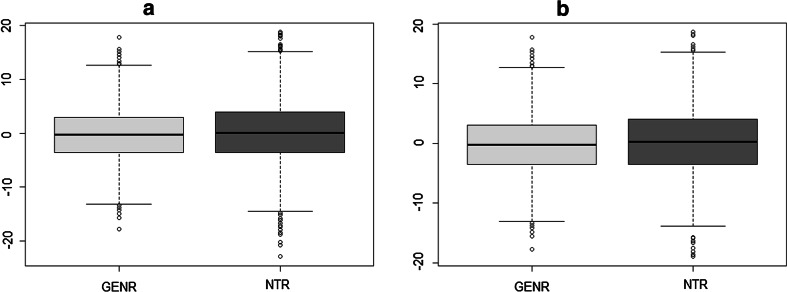
Distributions of height across cohorts after correction for age and sex. **a** Shows the distribution of height for all individuals. **b** Shows the distribution of height for the distantly related individuals

Two population based cohorts comprising a Dutch children supplied genotype information and data on height (Silventoinen et al. 2007; Jaddoe et al. 2012; Boomsma et al. 1992). Genotype data were available for 3102 children from the NTR and 2826 children from GENR (Table 1). All children were of Northwestern European Dutch background as was checked by PCA. Among them, 2226 subjects had height measurements in GENR and 2072 in NTR (Table 2; Fig. 2). After applying a cut-off of 0.025 for genetic relatedness recommended in GREML analyses (Yang et al. 2010) there were 1134 and 1990 individuals left in NTR and GENR, respectively, with height measurements. The NTR cohort comprised 528 males and 606 females at ages 4.6–11 years old. The GENR cohort comprised 998 males and 992 females at ages 4.8–9 years old (Table 3; Fig. 2). All parents gave informed consent. Study protocols were approved by Medical Ethics Committee of the VU University Medical Center, Amsterdam for NTR and by Medical Ethical Committee of the Erasmus Medical Centre, Rotterdam for GENR.

**Table 1:** Cohort description

Sample	N	Sex		N families	N independent observations
		Males	Females		
GENR	2826	1450	1376	171	2508^a^
NTR	3102	1381	1721	1709	1644^a^

^a^Based on the list of distantly related individuals, which were selected using GCTA cut-off 0.025 independently in each cohort

**Table 2:** Height measurements of all individuals

Sample	N	Sex		Age mean (SD)	Height in centimeters mean (SD)
		Males	Females		
GENR	2226	1124 (50.5 %)	1102 (49.5 %)	6 (0.4)	119.6 (5.6)
NTR	2072	948 (45.8 %)	1124 (54.2 %)	7.7 (1.4)	129.6 (9.8)

**Table 3:** Height measurements of the distantly related individuals

Sample	N	Sex		Age mean (SD)	Height in centimeters mean (SD)
		Males	Females		
GENR	1990	998 (50.2 %)	992 (49.8 %)	6.1 (0.4)	119.6 (5.6)
NTR	1134	528 (46.6 %)	606 (53.4 %)	7.7 (1.4)	129.7 (9.8)
GENR + NTR	3124	1526 (48.8 %)	1598 (51.2 %)	6.7 (1.2)	123.2 (8.8)

### Within sample pre-imputation SNP QC

The 3107 subjects in the NTR cohort were genotyped for 692,694 SNPs on Affymetrix 6.0 chip (Scheet et al. 2012). The 2830 subjects in the GENR cohort were genotyped for 489,878 SNPs on two Illumina chips (660 W, 610 K) (Medina-Gomez et al. 2015). Outliers were excluded from the GENR sample (4 individuals) and from the NTR sample (5 individuals) based on visual inspection of PC1 versus PC2 plots prior to analysis. As a result, individuals cluster within −0.06 > PC1 < 0.05 and −0.05 > PC2 < 0.07 intervals in GENR and −0.06 > PC1 < 0.06 and −0.05 > PC2 < 0.04 intervals in NTR. For GENR, the overlapping SNPs between the two platforms were used as input for imputation as reported before (Benke et al. 2014). Standard quality control steps were applied to the separate data sets using Plink 1.07 (Purcell et al. 2007). A sample call rate >0.975 and a SNP call rate >0.950 were applied for both cohorts. SNPs with minor allele frequency (MAF) <0.001 and SNPs with Hardy–Weinberg equilibrium (HWE) *p* value <10^−5^ were excluded. Individuals were checked for excess heterozygosity and subjects with an inbreeding coefficient, as estimated in Plink, F ≤ −0.05 or F > 0.05 were excluded. Identical by state (IBS), identical by descent (IBD) and gender mismatch were checked and samples not fitting the expected relations and/or gender were removed.

The next quality control step was a cross-check of alleles and SNP positions between the two cohorts as well as the GoNL reference set v.4 (build 37). SNPs that did not match by strand were flipped to the reference set strand. SNPs with discordant alleles or that were not present in the reference set were excluded. Genotyped data from the NTR and GENR cohorts have 120,568 overlapping autosomal SNPs, of which 255 (0.2 %) SNPs were significantly different in frequency across cohorts (*p* value <10^−5^, one-sided test). Pairwise comparison between the SNPs overlapping in NTR and GoNL, in GENR and GoNL and in NTR and GENR combined identified 4001 SNPs, which were significantly different in allele frequency (*p* value <10^−5^, 1969 between NTR and reference set, 2012 between GENR and reference set and 255 between NTR and GENR combined). All SNPs differing in allele frequency were removed. The resulting set of SNPs was either present on both platforms and in the reference set, or in a single platform and in the reference set. In order to minimize the amount of imputation stratification between samples, we selected the SNPs from the GoNL reference set that were present either on one or both genotyping platforms (Illumina or Affymetrix, N = 989,757) using VCFtools (Danecek et al. 2011).

After QC was performed there were 3102 NTR (1381 males, 1721 females) and 2826 GENR (1450 males, 1376 females) individuals left. These individuals were genotyped for 641,554 and 468,259 SNPs in NTR and GENR respectively. The two data sets were merged in Plink for pre-combined imputation.

### Imputation strategies

First explorations of pre-combined cross-platform imputation approaches were done for chromosome 22. Genotype data comprising 13,712 SNPs were extracted, phased and imputed using the three methods described below, aiming to determine the one to apply to the autosomal genome. The first approach uses MaCH phasing (selected because GCTA can read MaCH dosage files) and, inherently, also imputation of the missing genotypes. No reference set is involved. The second approach uses MaCH phasing but this time with the GoNL reference set. Here the haplotypes are predicted and genotypes are imputed based on the GoNL reference set, which contains the full SNP haplotypes representing the Dutch population regardless of the platform. The third approach uses MaCH-Admix instead of MaCH. Here, a new piecewise reference selection method is employed (Liu et al. 2013) with GoNL as a reference set. This method, which is implemented in MaCH-Admix, breaks a genomic region into small pieces and searches for haplotypes in the reference set that matches every piece. In all three approaches we imputed missing genotypes as dosage scores. We have not considered only using the SNPs that were present on both platforms, because the final data set would comprise of only ≈120 K SNPs after genome-wide QC.

After an imputation approach for the pre-combined dataset is chosen, we evaluate the effect of the two possible scenarios of imputation on platform stratification and SNP-heritability estimates. In the first case we pre-combine datasets and then impute using chosen approach; in the second case we impute datasets independently using the same software and reference set as for pre-combined dataset and post-combine.

### Post-imputation SNP QC

Post imputation QC aimed to examine the stratification between NTR and GENR due to genotyping platform after imputation on chromosome 22 at first and on the autosomal genome afterwards. A comparison between all imputation approaches was done based on the imputation quality metric (R^2^) calculated by the MaCH tools. The R^2^ measures imputation quality and ranges between 0 and 1 with higher value indicating better imputation accuracy, hence better genotype prediction. We used R^2^ to inspect whether filtering on this measure helps to reduce platform stratification. Subsequently, a case–control analysis of the imputed sample with cohort as phenotype was done using the Mach2dat software (Li et al. 2010) for dosages and Plink for best-guess to check if there were differences in allele frequencies after imputation. Note that in order to pool two independently imputed samples we had to (1) convert dosage files to best-guess and (2) merge using Plink. The latter should be taken into account when comparing N of SNPs different in frequency between cohorts based on dosages and best-guess. The threshold for significance chosen was a genome-wide suggestive *p* value of 10^−5^.

### Genetic pairwise relationships estimation (GRM)

Genetic relationship matrices (GRMs) were built from pre-combined cross-platform imputed dosages of the three approaches for chromosome 22 using GCTA. Different SNP filter criteria can be used to build these GRMs, which might affect the results. Therefore, we employed the criteria from three filters to estimate the matrices resulting in 9 GRMs. These criteria were: (1) without any filtering options on SNPs, (2) filtering on the imputation quality of R^2^ > 0.8, leaving only the high quality imputed SNPs and (3) filtering with R^2^ > 0.8 and MAF > 0.01, additionally excluding alleles with low minor allele frequency. To estimate the effects of stratification by SNP platforms after imputation we examined the GRMs using PCA in GCTA tool. We performed PCA on data from unrelated individuals. As PCs can be confounded by inversions of long linkage disequilibrium (LD) regions of chromosomes, which are observed in the Dutch population (Price et al. 2008; McEvoy et al. 2009), we pruned GoNL for LD with standard Plink options (--indep 50 5 2), excluded 24 long LD regions (Abdellaoui et al. 2013) and repeated PCA for each GRM selecting GoNL pruned set of SNPs. The method that showed the least stratification due to genotyping platform and higher imputation quality was chosen for the pre-combined cross-platform imputation of the autosomal genome. To explore the effect of cross-platform imputed pre-combined, cross-platform imputed post-combined and combined GRMs on SNP-heritability estimate of childhood height, we built: (1) a GRM with MAF > 0.01 and R^2^ > 0.8 filters from the total cross-platform imputed data set, (2) a GRM with MAF > 0.01 and R^2^ > 0.8 filters from NTR and GENR cohorts imputed independently and (3) a GRM with a MAF > 0.01 from QC-ed NTR and GENR genotypes combined, merged in Plink. Additionally, to check the effect of QC we built the GRM with MAF > 0.01 and R^2^ > 0.8 filters from the total cross-platform imputed data set excluding SNPs significantly different in frequency between cohorts after imputation. To distinguish between combination approaches throughout the paper we will refer to these GRMs as “imputed”, “imputed independently”, “combined” and “imputed clean”, respectively. Finally, SNP-heritability of height was estimated in NTR and GENR after building two separate GRMs with MAF > 0.01 filter from QC-ed NTR and GENR samples. We performed PCA for each of the autosomal GRM based on GoNL pruned set of SNPs and included these PCs in the analysis of height.

### Statistical analysis

#### Estimation of variance due to genetic effect of childhood height

Using GCTA, we estimated SNP-heritability of height using GRMs based on the autosomal genome. Imputation, SNP quality control as well as employing the different imputation approaches all determine the GRM relatedness of individuals. Therefore, for fair comparison between different ways of combing the genotype data in a GRM, we used the same unrelated individuals for each analysis. These were selected using the relatedness cut-off of 0.025 for individuals with height measurements from the combined and imputed GRMs (N = 3124). The difference in relatedness selection between the combined and imputed GRM was 22 individuals, which were excluded from the analyses. For the independent study analyses, however, we selected unrelated individuals, as one would have based on the GRM of the single study alone, using the same GRM cut-off of 0.025. Hence, if there are samples with family relations between NTR and GENR studies, they are still included in these separate study analyses.

In the SNP-heritability analyses, age and sex were included as covariates. To test whether there is still a platform effect present after imputation we included cohort as an extra covariate in addition to sex and age and compared results of both analyses. To detect and account for possible genetic stratification in relation to height (Abdellaoui et al. 2013) we included the first 10 PCs obtained from each GRM for unrelated individuals excluding long LD regions. Finally, we ran association analysis of height for imputed, combined, NTR and GENR datasets, with age and sex as covariates for unrelated individuals and built quantile-quantile (QQ) plots to check for possible inflation of the test statistics before and after pooling cohorts together without using 10 PCs and cohort as covariates.

## Results

### Imputation method

**Fig. 3:**
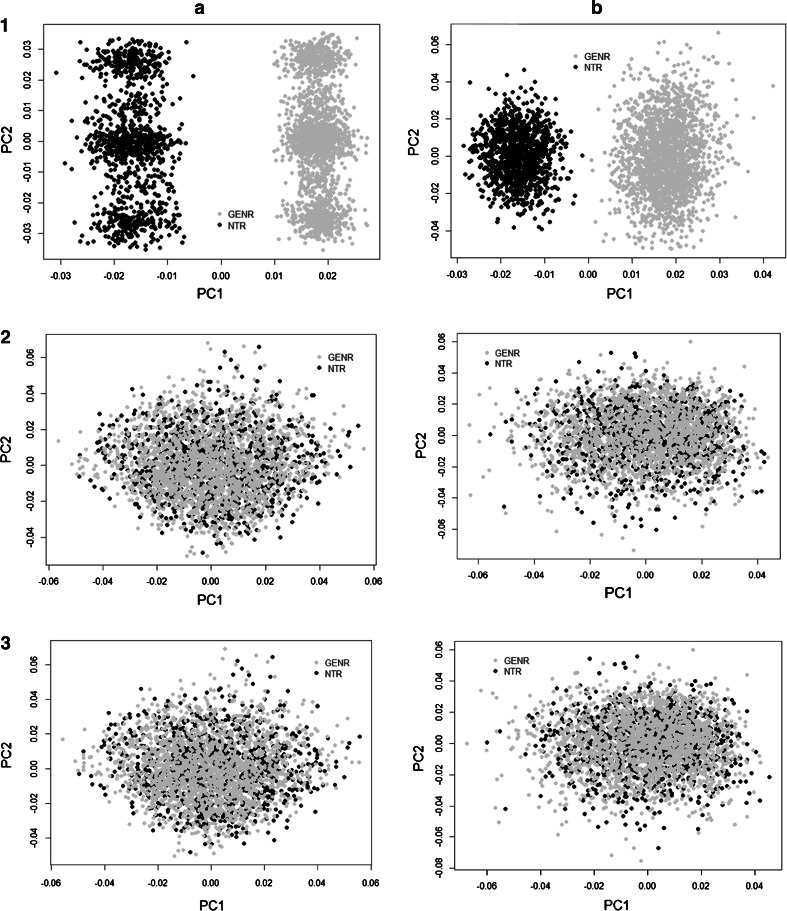
Comparison of imputation quality for chromosome 22. **1**–**3 (a, b)** PC1 versus PC2 plots of GRM based on MaCH without reference set, MaCH with reference set and MaCH-Admix with reference set respectively. **a**, **b** PCs plots including and excluding long LD regions (**a**. including, **b**. excluding). All PC plots are based on GRMs filtered with R^2^ > 0.8 and MAF > 0.01, where* black color* represents NTR and *grey color* represents GENR

**Fig. 4:**
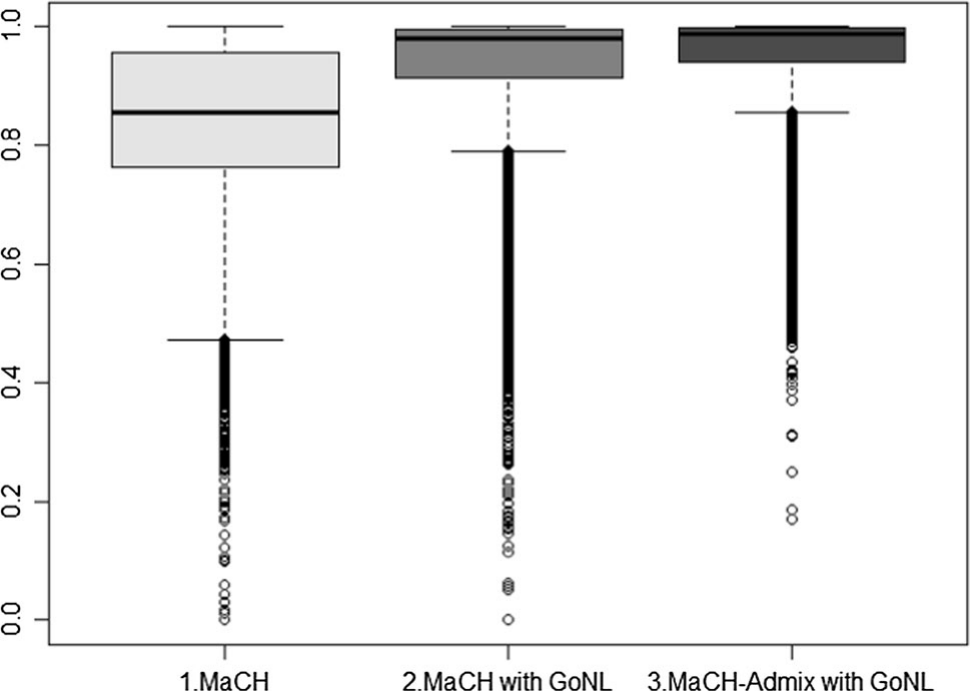
Comparison of R^2^ distribution of three methods for chromosome 22

Three imputation approaches aimed to pre-combine and cross-platform impute two cohorts were tested on chromosome 22: the first was MaCH without a reference set (i.e., the two datasets were only phased and imputed against each other), the second was MaCH with the GoNL reference set and the third was MaCH-Admix with the GoNL reference set. The comparison of the post-imputation quality control measures for these approaches is shown in Figs. 3 and 4. A NTR versus GENR case–control analysis after imputation showed that 4535, 203, and 93 SNPs were significantly different in frequency for the first, second and third method, respectively (*p* < 10^−5^, Wald test). The R^2^ measure also demonstrated different imputation quality: mean = 0.83 and median = 0.86 for the first, mean = 0.93 and median = 0.98 for the second and mean = 0.95 and median = 0.99 for the third method.

**Fig. 5:**
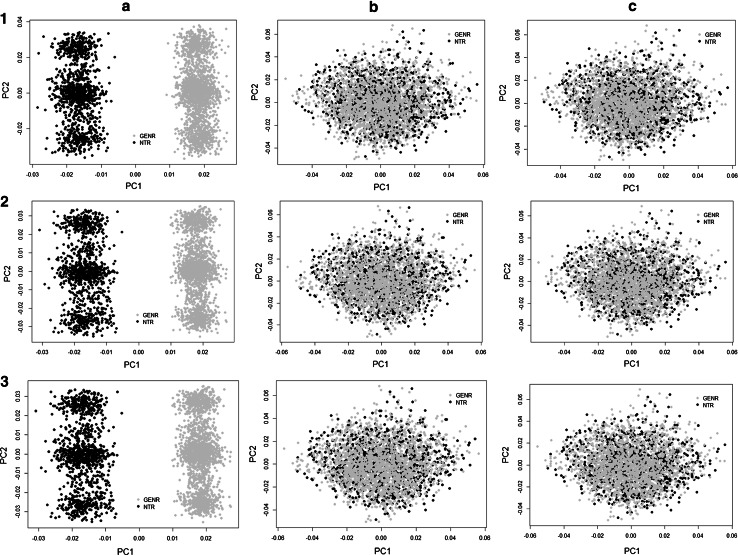
Chromosome 22 PC plots based on GRMs, each with three filtering options. **a** (**1**–**3**) the performance of MaCH without reference set, **b** (**1**–**3**) the performance of MaCH with reference set and **c** **(1**–**3**) the performance of MaCH-Admix with reference set. **1**–**3** application of different filter criteria (1. none, 2. R^2^ > 0.8, 3. R^2^ > 0.8 and MAF > 0.01) for the corresponding imputation method

We plotted the first (PC1) and second (PC2) principal components for each imputed GRM matrix in R (Team RC 2012). In Fig. 3 the GRMs based on the R^2^ > 0.8 and MAF > 0.01 filters are shown. As expected given the median quality of SNPs, filtering on R^2^ and MAF (4611 and 46, 1684 and 106, 1186 and 105 SNPs were excluded in the first, second and third approach, respectively) did not affect the outcome of the imputation results (Fig. 5). As shown in Fig. 3(1a), PC1 clearly captures the cohort differences due to genotyping platform. GENR and NTR are separated into two clusters with the first PC. For the PC2 component we observe three blocks that disappear after eliminating the long LD regions as shown on Fig. 3(1b). Figures 3(2a, 2b) show that homogeneity is reached when using MaCH phasing with a reference set, with and without excluding long LD regions. Similarly, Figs. 3(3a, 3b) using MaCH-Admix instead of MaCH also shows no population stratification due to genotyping platform. Finally, from Fig. 4 it becomes clear that MaCH-Admix outperforms MaCH with overall imputation quality.

When examining imputation differences for individual SNPs by comparing the allele frequencies between cohorts, we identified some significantly different SNPs, as was noted above. We computed squared LD correlations between each significant SNP that resulted from post-imputation QC analysis of the chromosome 22 imputation with MaCH-Admix and all neighboring SNPs within a 1 Mb region in Plink. The majority of these estimates were low (interquartile range = 0.0009, mean = 0.005, median = 0.0003), indicating regions with weak LD around significant SNPs. Therefore we can hypothesize that these SNP differences may arise from imperfect phasing and imputation for these SNPs with low LD.

**Fig. 6:**
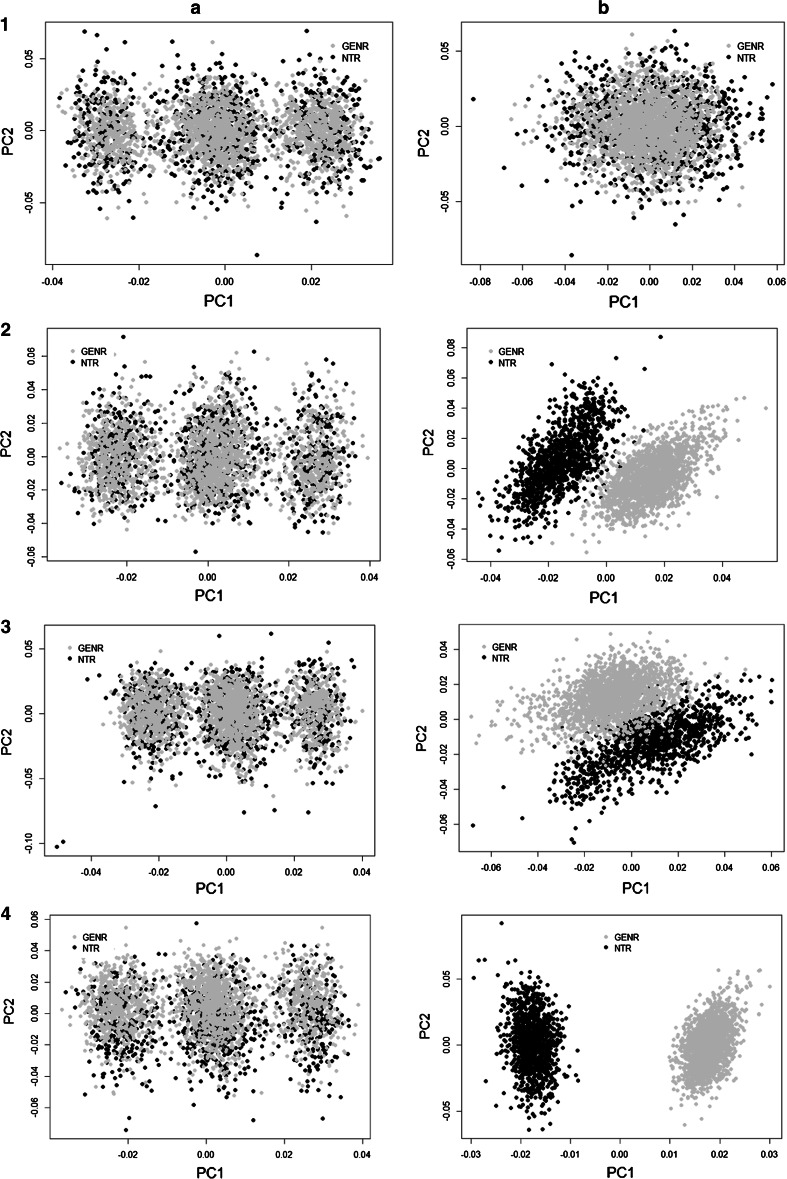
PCA results of combined (**1a**, **b**), imputed (**2a**, **b**), imputed clean (**3a**, **b**) and imputed independent datasets (**4a**, **b**), respectively. PC1 versus PC2 plots are made from GRM with R^2^ > 0.8 and MAF > 0.01 filters in case of imputed and with MAF > 0.01 filters in case of combined GRMs. **a**–**b** Shows PCs plots including and excluding long LD regions (**a**. including, **b**. excluding)

**Fig. 7:**
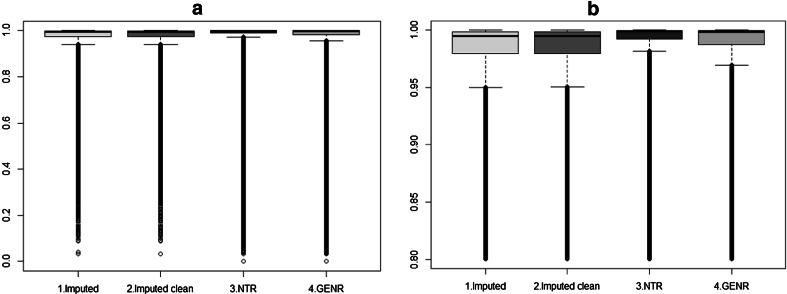
Comparison of R^2^ distribution of imputed, imputed clean, independently imputed NTR and GENR datasets. **a** all SNPs, **b** SNPs with R^2^ > 0.8

**Fig. 8:**
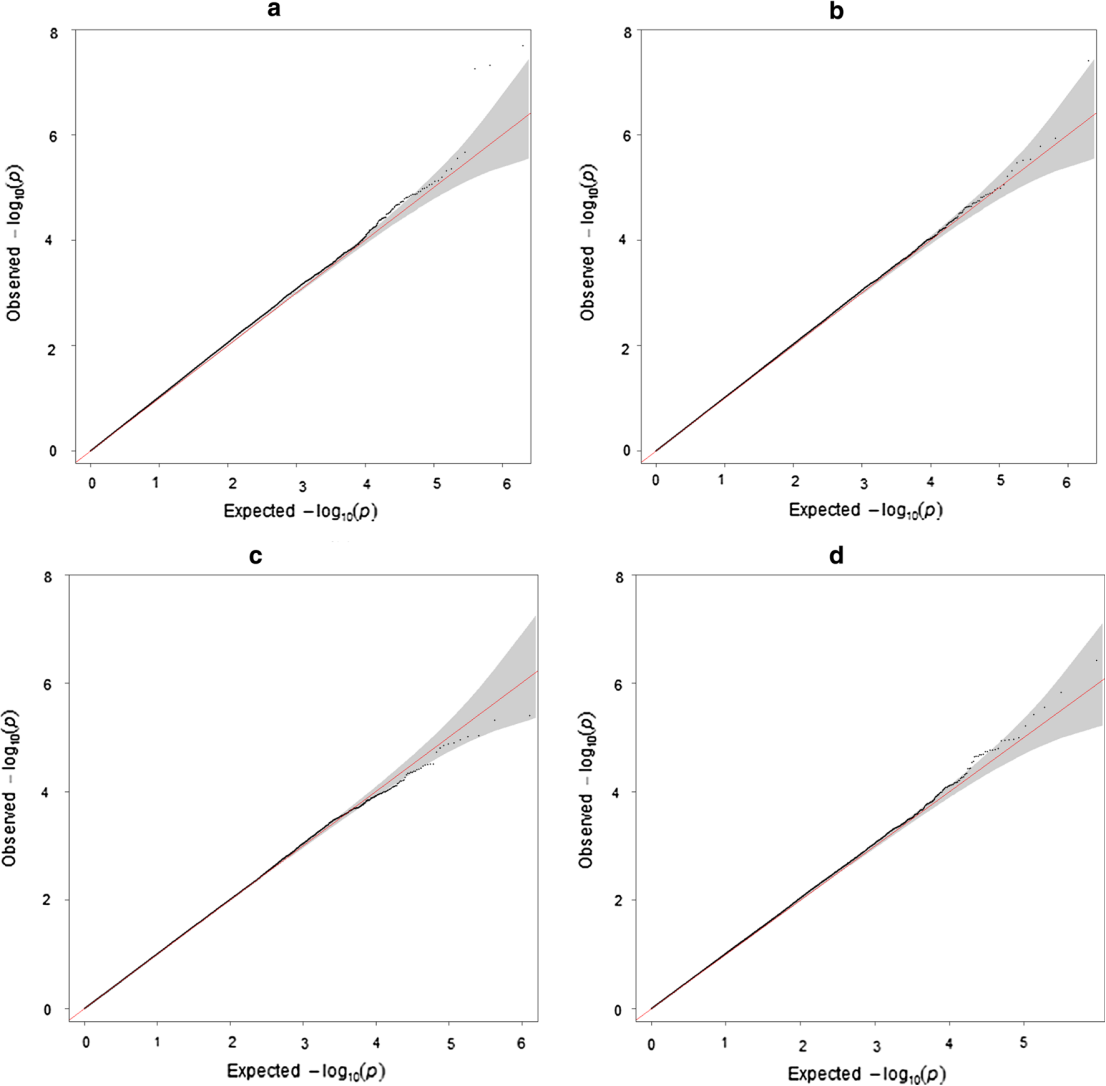
Quantile-quantile plots based on test-statistics from association analysis of height of **a** imputed, **b** combined, **c** NTR and **d** GENR datasets respectively

Repeating the same MaCH-Admix imputation procedure of chromosome 22, (1) the NTR and GENR pre-combined sample was cross-platform imputed for all autosomal chromosomes and subsequently an “imputed” GRM was made; (2) the NTR and GENR samples were imputed independently for all autosomal chromosomes, post-combined and an “imputed independently” GRM was built. Figures 6 and 7 demonstrates QC results after imputation of the whole sample: Fig. 6 shows PC1 and PC2 plot with and without exclusion of long LD regions and Fig. 7 displays the R^2^ distribution for imputed (mean = 0.97, median = 0.99), imputed clean (mean = 0.97, median = 0.99), NTR imputed independently (mean = 0.97, median = 1.0) and GENR imputed independently (mean = 0.96, median = 1.0) samples. The quality of imputation in NTR seems slightly better than in GENR, which showed 203 monomorphic SNPs after imputation. These SNPs were excluded from calculation of mean and median of R^2^ for GENR. They also did not contribute to further analysis, as they have MAF = 0 and were filtered out with MAF > 0.01 option. As shown in Fig. 6(1a–4a) PC2 captures three blocks that are inversions of long LD regions of chromosomes and we do not observe any cohort differences due to the genotyping platform for any of GRMs resulted after different combination approaches. After exclusion of long LD regions, PC1 and PC2 capture population structure for each of the approaches (Fig. 6(1b to 4b). Figure 8 displays QQ plots of GWAS test-statistics for imputed (lambda (λ) = 1.04), combined (λ = 1.02), NTR (λ = 1.01) and GENR (λ = 1.02) datasets. NTR versus GENR case–control analysis showed a total of 4340 SNPs and 18,306 SNPs that significantly differ in frequency after imputation, when datasets were pre-combined and imputed and imputed and post-combined, respectively. We excluded 4430 SNPs from GRM “imputed” to build GRM “imputed clean”.

### Heritability of childhood height

The pooled data set comprised a total of 3124 distantly related individuals, where 1526 were males and 1598 were females. Childhood mean height in the pooled data set was 123.2 cm (SD = 8.8) at mean age of 6.7 years (SD = 1.2) (Table 3). GREML analysis of height yields a SNP-heritability estimate of 0.43 (SE = 0.10) when combining (not imputing) the data from both cohorts (Table 4). The estimates of the SNP-heritability based on GRMs of the imputed data are 0.51 (SE = 0.10), and 0.49 (SE = 0.10) after cleaning SNPs that were significantly different between the two cohorts. The estimate of the SNP-heritability based on GRM data imputed independently is 0.52 (SE = 0.10). When considering only NTR individuals or GENR participants in the various GRM matrices, NTR gives estimates of 0.42 (SE = 0.29), 0.39 (SE = 0.29), 0.45 (SE = 0.29) and 0.50 (SE = 0.28) for the imputed GRM, imputed clean GRM, imputed independently and combined GRMs, respectively; GENR gives estimates of 0.52 (SE = 0.16), 0.52 (SE = 0.16), 0.53 (SE = 0.16), 0.58 (SE = 0.17) for the imputed GRM, imputed clean GRM, imputed independently and combined GRMs, respectively. The variances explained by the independent cohorts were 0.47 (SE = 0.27) for NTR and 0.57 for GENR (SE = 0.17), if one would conduct two separate GCTA studies. These results show that for each of the individual cohorts (NTR or GENR)—selected either from the imputed GRMs or from combined—the amount of variance explained by the SNPs remains the same given the large standard errors. Strikingly, cross-platform imputed GRMs shows suggestive, if any, increase of the variance explained by the SNPs in comparison to the combined (not imputed) GRM. If cohort is taken into account as a covariate, results show a ≈2 % reduction of explained variance in the cross-platform imputed GRMs, while the combined GRM estimate remains the same (Table 5). This indicates that there is still little stratification left by platform. Repeating the comparison procedure including the first 10 PCs resulted in SNP-heritability estimates that were on average ≈11 % lower for all pooled GRMs, ≈13 % for NTR and ≈7 % for GENR (Table 6). When cohort was used as a covariate together with 10 PCs (Table 7) there was no effect on SNP-heritability estimates in comparison to the effect of 10 PCs alone. The comparison of results shows that all SNP-heritability estimates, given the standard errors, are not significantly different from each other. However, the standard errors are largely reduced as the sample size increased by combining the two cohorts allowing the SNP-heritability to reach significance.

**Table 4:** SNP-heritability (h^2^) results of analyses of height based on imputed, imputed clean, imputed independently and combined GRMs including results of specific analysis of NTR and GENR selected individuals

Data set	h ^2^	SE	N	*P* value
Imputed^a^	0.51	0.10	3124	1 × 10^−7^
Imputed clean^b^	0.49	0.10	3124	2.9 × 10^−7^
Imputed independently^c^	0.52	0.10	3124	8.8 × 10^−8^
Combined^d^	0.43	0.10	3124	2 × 10^−6^
NTR imputed^a^	0.42	0.29	1134	0.07
NTR imputed clean^b^	0.39	0.29	1134	0.09
NTR imputed independently^c^	0.45	0.29	1134	0.07
NTR combined^d^	0.50	0.28	1134	0.04
NTR independent^e^	0.47	0.27	1173	0.04
GENR imputed^a^	0.52	0.16	1990	3.7 × 10^−4^
GENR imputed clean^b^	0.52	0.16	1990	3.9 × 10^−4^
GENR imputed independently^c^	0.53	0.16	1990	3.4 × 10^−4^
GENR combined^d^	0.58	0.17	1990	2 × 10^−4^
GENR independent^e^	0.57	0.17	1994	2.2 × 10^−4^

^a^GRM based on data cross-platform imputed SNPs

^b^GRM based on data cross-platform imputed SNPs, excluding SNPs significantly different in frequency

^c^GRM based on SNPs imputed separately and combined afterwards

^d^GRM based on the combined SNP data without imputation

^e^GRM based on each genotyped sample separately

**Table 5:** SNP-heritability (h^2^) results of analyses of height with cohort included as a covariate based on imputed, imputed clean, imputed independently and combined datasets

Data set	h ^2^	SE	n	*P* value
Imputed^a^	0.49	0.10	3124	3 × 10^−7^
Imputed clean^b^	0.47	0.10	3124	7 × 10^−7^
Imputed independently^c^	0.50	0.10	3124	3.6 × 10^−7^
Combined^d^	0.43	0.10	3124	3.8 × 10^−6^

^a^GRM based on data cross-platform imputed SNPs

^b^GRM based on data cross-platform imputed SNPs, excluding SNPs significantly different in frequency

^c^GRM based on SNPs imputed separately and combined afterwards

^d^GRM based on the combined SNP data without imputation

**Table 6:** SNP-heritability (h^2^) results of analyses of height based on imputed, imputed clean, imputed independently and combined datasets adjusted for age, sex and 10 PCs, but not for cohort as covariate. Additionally, results of analysis of height in NTR and GENR independent cohorts adjusted for age, sex and 10 PCs

Data set	h ^2^	SE	N	*P* value
Imputed^a^	0.41	0.11	3124	4.6 × 10^−5^
Imputed clean^b^	0.38	0.11	3124	1.2 × 10^−4^
Imputed independently^c^	0.39	0.11	3124	1.2 × 10^−4^
Combined^d^	0.33	0.10	3124	7.2 × 10^−4^
NTR independent^e^	0.34	0.28	1173	0.12
GENR independent^e^	0.50	0.17	1994	1.6 × 10^−3^

^a^GRM based on data cross-platform imputed SNPs

^b^GRM based on data cross-platform imputed SNPs, excluding SNPs significantly different in frequency

^c^GRM based on SNPs imputed separately and combined afterwards

^d^GRM based on the combined SNP data without imputation

^e^GRM based on each genotyped sample separately

**Table 7:** SNP-heritability (h^2^) results of analysis of height based on imputed, imputed clean, imputed independently and combined datasets adjusted for age, sex and 10 PCs, as well as for cohort as covariate

Data set	h ^2^	SE	N	*P* value
Imputed^a^	0.41	0.11	3124	5 × 10^−5^
Imputed clean^b^	0.38	0.11	3124	1.4 × 10^−4^
Imputed independently^c^	0.39	0.11	3124	1.2 × 10^−4^
Combined^d^	0.32	0.10	3124	9 × 10^−4^

^a^GRM based on data cross-platform imputed SNPs

^b^GRM based on data cross-platform imputed SNPs, excluding SNPs significantly different in frequency

^c^GRM based on SNPs imputed separately and combined afterwards

^d^GRM based on the combined SNP data without imputation

## Discussion

GREML estimates the narrow-sense heritability from all common SNPs genotyped or imputed in a sample. However, often sample sizes are small, for example, when closely related individuals are excluded. In this paper, we examined imputation-phasing approaches to create a GRM that combines genotype data across genotype platforms and cohorts and explored the effect of using different GRM build strategies, when cohorts are (1) just combined, (2) pre-combined and cross-platform imputed and (3) cross-platform imputed and post-combined (Fig. 1). Imputed GRM genetic relationships between individuals are estimated within studies as well as between studies based on all Illumina and Affymetrix SNPs. Combined GRM genetic relationships are estimated in three groups: the within cohort pairs of NTR which all have Affymetrix SNPs, the within cohort pairs of GENR which all have Illumina SNPs, and the between cohort pairs which only have the overlapping SNPs. Therefore cross-platform imputation is required to supply individuals genotyped on one platform with SNPs genotyped on another platform. Note that we do not aim to impute a large number of additional (rare) SNPs from the reference set to increase number of SNPs. Instead the total number of SNPs in cross-platform imputed dataset remains approximately the same (Affymetrix SNPs + Illumina SNPs), but all individuals from both cohorts pooled together have complete information from the same SNPs. In this way we tried to minimize the possible differences between platforms, while also trying to retain as much information of the genotyping platforms as possible. Because the quality of cross-platform imputation depends on LD-phase information, that correctly represents the Dutch population, from which GENR and NTR cohorts were drawn, we used the Dutch GoNL reference set.

Based on the chromosome 22 analyses of pre-combined cross-platform imputation approaches, we showed that phasing and imputation of missing genotypes with a reference dataset that contains all SNPs and LD information between these SNPs does not substantially increase cohort stratification due to genotyping platform within the GRM, while phasing without a reference set, lacking this essential LD information, does. Using only the SNPs that are overlapping between genotyping platforms as an imputation backbone is insufficient which was evident from the subsequent PC analyses. Given that one could consider two cohorts with different platforms as a stratified population, the use of MaCH-Admix additionally seems to have helped to improve the imputation quality. However, this effect was much weaker in comparison to the use of a reference set. The analysis based on PCs, also showed that post imputation filtering on MAF and R^2^ did not largely seem to influence the cohort stratification, mainly because the quality of the imputed SNPs was generally high. Imputation of the autosomal genome followed by PC analysis showed that to some extend there is still platform stratification present after imputation (Fig. 6). Interestingly, the combined GRM did not show platform stratification, which may indicate that a backbone of ≈120 K SNPs is enough to estimate the genetic relationships between individuals from different cohorts.

The analysis of childhood height yielded relatively the same estimates of SNP-heritability for cross-platform imputed GRMs, suggesting a slight increase of the estimate in comparison to the combined GRM. Adjusting for 10 PCs with or without study as covariate results in ≈11 % reduction of SNP-heritability for all GRMs, including the combined one. Whereas there was only ≈2 % reduction in SNP-heritability when study was used as a covariate for imputed GRMs and not for the combined one. PC adjustment of independent cohorts results in a SNP-heritability drop of ≈13 % for NTR and ≈7 % for GENR. Drop in NTR SNP-heritability estimate in contrast to GENR is more pronounced, as individuals in NTR spread across the Netherlands resulting in a more diverse cohort. Given that λ estimates obtained from association analysis are not inflated it is possible that PCs may capture true variation of height along with platform stratification and may overcorrect the estimates. On the other hand, PCs may help to capture and correct for other sources of stratification within cohorts. Interestingly, SNP-heritability estimates resulting from GRM imputed and GRM imputed independently are approximately the same for all conditions. Moreover, SNP-heritability estimates from the combined GRM are just slightly lower in comparison to the imputed GRMs, which may support the conclusion that relationships between individuals across cohorts, estimated from SNPs overlap of ≈120 K, is enough to explain substantial proportion of variation in childhood height.

In this study we estimated SNP-heritability of childhood height using different GRM building strategies. These GRMs yielded significant estimates of SNP-heritability in range from 0.33 to 0.52 depending on various correction options. Height is a highly heritable trait with heritability estimates ranging from 0.89 to 0.93 in adults (Silventoinen et al. 2003). A SNP-heritability of 60 % has been estimated based on all common SNP together in the recent GWA meta-analysis study of adult height (Wood et al. 2014). In children, heritability estimates vary during growth. Mook-Kanamori et al. showed that heritability increases from 26 and 27 % at birth to 63 and 72 % at 36 months in twins from the NTR study and in singletons from GENR study (parent–child trio’s design) (Mook-Kanamori et al. 2012). Notably, heritability estimates for singletons and twins were very similar, justifying the pooling of data from these cohorts. In this study we have used height, which is a highly heritable GCTA benchmark trait and it can be easily measured. For other traits, which are less heritable and less easily measured additional increase of sample size may be required in order to increase power to accurately estimate SNP-heritability. To calculate the power given a sample size one can use the GCTA-GREML Power Calculator (Visscher et al. 2014).

Strategies aiming to detect and correct for platform stratification after cross-platform imputation were considered in this study for cohorts with the same ethnicity. However, when combining cohorts with a different ethnicity this approach is unlikely going to be appropriate for several reasons (de Candia et al. 2013). First, SNP-heritability of combined multi-ethnic dataset will depend on the heritability of the trait in each population, which can differ. Second, different LD-patterns may imply that causal SNPs in one population will be tagged better than in the other population. Third, if cohorts with different ancestry are genotyped on different platforms it might be difficult to distinguish the two confounding factors, platform and population stratification. Finally, informative SNPs that are common in one population and rare in another will be eliminated from analysis after QC and effect of remaining SNPs, reflecting ancestry, will be corrected with PCs. Thus, the estimate would reflect part of SNP-heritability, which is based on causal SNPs shared across ethnicities. The extent to which causal SNPs are shared between different ethnicities depends on the genetic architecture of the trait in each population. For example, a recent study has provided an evidence that genetic variation is largely shared between two different ethnic cohorts, African and European, for schizophrenia risk (de Candia et al. 2013). There are also other statistical methods that can be applied to combine cohorts information to estimate the SNP-heritability of traits, such as the density estimation (DE) method (So et al. 2011). The DE method does not require the raw genotype data, as it uses summary statistics from GWAS or meta-analysis GWAS. However, it requires LD-pruning to obtain a list of relatively independent SNPs to estimate their effect, which may result in variability of estimates depending on the pruning threshold and on SNP density in a single GWAS (van Beek et al. 2014). Van Beek et al. also suggested that SNP-heritability can be underestimated due to genotypic heterogeneity or phenotypic differences between cohorts in meta-analysis GWAS and summary statistics correction, such as for multiple testing and genomic control inflation factor.

In conclusion, using the complete information of a reference set for phasing and imputation of all SNPs on two different genotyping platforms, allows the combination of cohort data genotyped on both of these platforms. When combining genotype data across platform or cohort thorough pre- and post QC is required, which can be tested with association and principal component analyses. For our approach we assume that the cohorts have a similar ethnicity/genetic background. To account for platform stratification or phenotypic differences in the dataset, cohort should always be included as a covariate. Whether one should use imputation, or just combine the genotype data, depends on the number of overlapping SNPs in relation to the total number of genotyped SNPs for both cohorts, and their ability to tag all the genetic variance related to the specific trait of interest.
